# Antiplatelet response to clopidogrel is associated with a haplotype in *CYP2C19* gene in Pakistani patients

**DOI:** 10.1038/s41598-022-09679-8

**Published:** 2022-04-13

**Authors:** Sagheer Ahmed, Saima Gul, Sami Siraj, Abrar Hussain, Fahad Sultan Sheikh, Saeed Ullah Shah, Kholood Janjua, Hizbullah Khan, Mohammad Hamid Hamdard

**Affiliations:** 1 Shifa College of Pharmaceutical Sciences, Shifa Tameer-e-Millat University, Islamabad, Pakistan; 2 Department of Physical Therapy, Shifa Tameer-e-Millat University, Islamabad, Pakistan; 3 Institute of Basic Medical Sciences, Khyber Medical University, Peshawar, Pakistan; 4 Department of Biotechnology & Informatics, Balochistan University of Information Technology, Engineering and Management Sciences, Quetta, Pakistan; 5 Department of Cardiology, Shifa International Hospital, Islamabad, Pakistan; 6 Mahroof International Hospital, Islamabad, Pakistan; 7 Faculty of Biological Sciences, Kabul University, Kabul, Afghanistan

**Keywords:** Cardiology, Health care, Medical research

## Abstract

Clopidogrel, an antiplatelet drug, is frequently prescribed to patients diagnosed with ischemic diseases such as those suffering from acute coronary syndromes or ischemic stroke. Despite the drug being effective in majority of the patients, some still experience ischemic events early in the treatment which might be due to poor platelet inhibition. This study aims to investigate the association of cytochrome P450 2C19 (*CYP2C19*) loss-of-function polymorphisms, haplotypes as well as a wide range of clinical and demographic variables with platelet aggregation phenotypes to clopidogrel in a Pakistani cohort. The study comprised of a total of 120 patients diagnosed with cardiovascular diseases and were treated with clopidogrel. Antiplatelet response to clopidogrel was monitored by Helena AggRAM (HL-2-1785P) and patients with maximal platelet aggregation more than 50% were categorized as low responders and those with less than 50% as high responders. Our results show that 56.6% of patients were homozygous for the *CYP2C19* wild-type allele, 38.3% of patients possessed one copy of the CYP2C19*2 allele and 5% of patients possessed both CYP2C19*2 alleles. No CYP2C19*3 allele was found in our patient cohort. There was no statistically significant difference between the high and low responder groups to clopidogrel in terms of extensive, intermediate and poor metabolizer genotypes. However, haplotype (H1), leukocyte count, random blood glucose, and history of diabetes mellitus was associated with the antiplatelet response to clopidogrel. The prevalence of clopidogrel resistance in our population was in line with that reported for other regional and global populations.

## Introduction

Clopidogrel, an antiplatelet drug which targets thienopyridine P2Y12 receptor, is frequently employed in patients who have been diagnosed with ischemic heart disease, ischemic stroke and acute coronary syndrome (ACS)^1–3^. Clopidogrel functions as an irreversible antagonist of the receptor and is effective in majority of the patients. However, a significant patient population with ACS who are concomitantly treated with aspirin and clopidogrel faces the recurrence of an acute ischemic event early in the treatment^4^. This ‘resistance’ to the therapeutic effect of clopidogrel might be due to the poor platelet inhibition by the drug which is likely to result in the poor therapeutic response to the antiplatelet therapy^5^.

Several studies investigating the clopidogrel ‘resistance’ report a prevalence of 15.9% to 49.5%, indicating a huge population-based variation^6^. This high variability among various populations suggests that environmental and genetic difference may play important roles in determining clopidogrel resistance. Genetic variation in the cytochrome P450*2C19* gene has been previously found associated with high ADP-induced platelet aggregation in clopidogrel treated patients^7,8^. However, it is unlikely that *CYP2C19* genetic variation is the only responsible factor for the observed clopidogrel resistance. Various environmental factors may also lead to clopidogrel resistance which may or may not be dependent on the bioavailability of clopidogrel^9,10^. The choice of blood preservative and the tests used to measure platelet aggregation also influence the clopidogrel’s antiplatelet response^11^.

Clopidogrel is absorbed in intestinal cells as prodrug, and then transported to hepatocyte where it is converted to active metabolite under the action of different isoforms of CYP enzymes, in a two-step oxidation. CYP2C19 is the principle contributor in both these steps^12^. Consequently, genetic variations in the *CYP2C19* gene bring about variations in the therapeutic responses of both patients and healthy volunteers to clopidogrel^13–17^. *CYP2C19* gene harbors up to 35 known variations or star alleles (*CYP2C19* *1–35) as per pharmacogene variation consortium or PharmVar^18^. In contrast to individuals possessing the wild-type *CYP2C19* allele, a reduction in the inhibition of platelet aggregation is observed in clopidogrel treated patients carrying a single or both copies of the defective *CYP2C19* allele^14^. These defective alleles are involved in the inter-individual variation with respect to clopidogrel response^13,19^. Phenotypic consequence of most of these alleles can range from reduced effect to complete functional loss. Among these, CYP2C19*2 and *3 are considered defining variations. CYP2C19*2 (rs4244285) was the first variant discovered that induces an aberrant splice site in exon 5 (c.681G>A)^20^ while CYP2C19*3 (rs4986893) leads to a termination codon in place of one for tryptophan (c.636G>A, p.W212X)^21^. The decreased anti-platelet activity post clopidogrel administration is due to reduced levels of its active metabolite in carriers of at least one of the low activity alleles, CYP2C19*2 or *3^22^. Furthermore, differential clopidogrel response is observed in populations with high prevalence of CYP2C19*2 allele^23^. An increased risk of heart attack, stroke or high mortality is likely in patients carrying reduced function alleles of *CYP2C19* due to their inability to completely convert clopidogrel to its active metabolite. Therefore, a suitable strategy of pharmacogenetic testing for *CYP2C19* genetic variants has been recommended for determining patients who may be subjected to a higher risk of adverse events^24^.

The principal objective of present study is to find out the prevalence of resistance to clopidogrel therapy in Pakistani patients who are taking clopidogrel for various indications and to investigate its relationship with CYPC19*2 and *3 variants and haplotypes. Impact of several other clinical and demographic variables was also explored.

## Results

The demographic and basic clinical data of the patients who participated in this study is provided in the Table 1. The average age of our patient cohort was 60 ± 11 years with the patients in the low clopidogrel response group being slightly younger (58 ± 12) than those in the high response group (61 ± 11). The magnitude of platelet aggregation was measured as a percentage, with patients having a maximal platelet response of 0–50% were categorized as “High” responders and those patients having a maximal platelet response of > 50% were regarded as “Low” responders to clopidogrel. Out of the 120 patients 82.5% showed a high level of platelet inhibition. About three fifth of the patients were male. Specifically, in the low responder’s group, 81% (n = 17) patients were male; while 55% (n = 55) male patients were present in the high responders group.

**Table 1:** Baseline and demographic characteristics of the investigated population.

Variable		Overall (n = 120)	Response to clopidogrel (%)		p-value
			Low (n = 21)	High (n = 99)	
Gender	Male	71	17 (81)	54 (55)	
	Female	49	4 (19)	45 (46)	0.025*
Age	(Mean ± S.D.)	60 ± 11	58 ± 12	61 ± 11	0.348
BMI	(Mean ± S.D.)	23.62 ± 3.46	24.18 ± 3.23	23.50 ± 3.51	0.417
Concomitant diseases and risk factors	Diabetes	52	12 (57)	40 (40)	0.160
	Hypertension	80	14 (67)	66 (67)	1.00
	Smoking	37	8 (38)	29 (29)	0.428
	Family history of coronary artery disease	74	14 (67)	60 (61)	0.604
Indication	Acute coronary syndrome	64	12 (57)	52 (53)	
	Coronary artery disease	19	2 (10)	17 (17)	
	Congestive cardiac failure	6	1 (5)	5 (5)	
	Ischemic heart disease	11	4 (19)	7 (7)	
	Unstable angina	20	2 (10)	18 (19)	0.398
Concurrent medication	Statin	77	11 (53)	66 (67)	0.215
	ACE inhibitors	3	0 (0)	3 (3)	0.558
	Low molecular weight heparins	57	10 (48)	47 (48)	0.990
	Beta blocker	21	2 (10)	19 (20)	0.290

*p-value ≤ 0.05.

Patients exhibited co-morbidities and various risk factors, mainly diabetes (43%), hypertension (67%), frequent cigarette smoking (31%) and a familial history of coronary artery disease (62%). The majority of the patients were prescribed clopidogrel for ACS (53%), followed by coronary artery disease (16%), ischemic heart disease (9%), and congestive cardiac failure (5%). The concurrent medications used by the patients include statins, 64% (n = 77), angiotensin converting enzyme (ACE) inhibitors 2.5%, (n = 3), clexane 47.5% (n = 57), and beta-blockers 17.5% (n = 21). Clopidogrel indication, the use of concurrent medications, or co-morbidities were found to have no significant association with platelet response to clopidogrel (Table 1).

In all those patients who were administered clopidogrel, the association between the antiplatelet response to clopidogrel and the various biochemical markers was investigated as provided in the Table 2. Multiple parameters such as prothrombin time, activated partial thromboplastin time, hemoglobin, leukocyte count, platelet count, random glucose, urea, creatinine, and fasting blood sugar were measured. Overall, no statistically significant association was observed between the high responders to clopidogrel and most of the studied parameters. However, a significant association was detected between the high responders to clopidogrel and total leukocyte count with high responders having significantly lower leukocyte count.

**Table 2:** Baseline laboratory characteristics of the investigated population.

Variable		Overall (n = 120)	Response to clopidogrel		p-value
			Low (n = 21)	High (n = 99)	
Laboratory findings	PT^a^ (prothrombin time)	12.0 ± 5.0	11.0 ± 5.0	12.0 ± 5.0	0.411^b^
	APTT^a^ (activated partial thromboplastin time)	30.0 ± 90.0	26.0 ± 10.5	30.0 ± 9.0	0.197^b^
	Hemoglobin^a^	12.7 ± 3.0	13.0 ± 3.0	12.47 ± 3.0	0.079^b^
	White blood cells^a^	10.3 ± 3.1	11.37 ± 4.61	10.05 ± 3.16	0.018*^b^
	Platelets^a^	219.0 ± 79.0	218.0 ± 77.5	220.0 ± 79.3	0.743^b^
	Random blood sugar^a^	157.0 ± 157.0	176.0 ± 182	140.0 ± 154.0	0.078^b^
	Urea^a^	41.0 ± 32.0	42.0 ± 28.0	40.0 ± 32.5	0.571^b^
	Creatinine^a^	1.3 ± 0.9	1.30 ± 0.63	1.40 ± 0.90	0.549^b^
	Fasting blood sugar^a^	110.0 ± 40.0	100.0 ± 35.0	110.0 ± 40.0	0.339^b^
CYP2C19*2 (3 groups)	CC	68	9 (43)	59 (60)	0.291^c^
	CT	46	10 (48)	36 (37)	
	TT	6	2 (10)	4 (4)	
CYP2C19*2 (2 groups)	CC	68	9 (43)	59 (60)	0.160^c^
	CT/TT	52	12 (58)	40 (41)	
Haplotype	H1	84	10 (48)	74 (75)	
	H2	22	5 (24)	17 (18)	0.475^c^

*p-value ≤ 0.05.

^a^Median and inter-quartile range calculated for non-normally distributed continuous variables (Normality tested by Shapiro–Wilk test).

^b^Mann–Whitney U test applied for non-normal data.

^c^Chi-square test (or Fisher exact test for expected count per cell less than 5).

**Figure 1:**
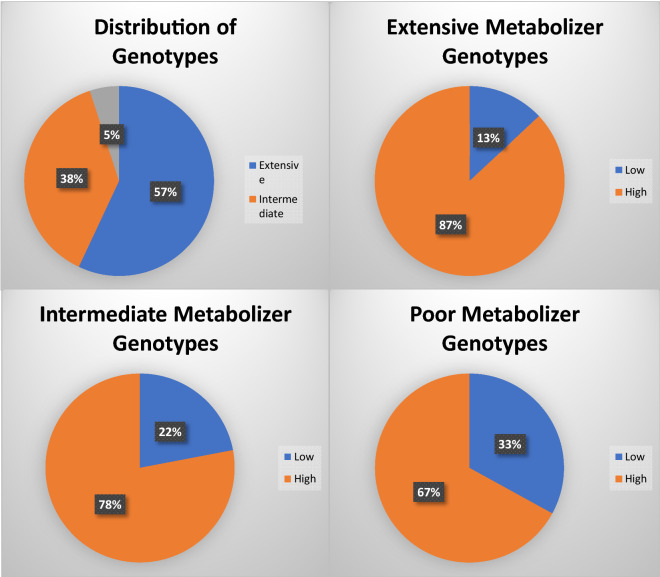
Genotype frequencies in our cohort, and low and high responders to clopidogrel in CYP2C19*1*1, *1*2, and *2*2 genotypes.

No CYP2C19*3 allele was observed in our cohort. The frequency of CYP2C19*2 allele was 0.241. The prevalence of the extensive metabolizer genotype (CC) in our patient cohort was 0.56 (n = 68), intermediate metabolizer genotype (CT) was 0.38 (n = 46), and poor metabolizer genotype (TT) was 0.05 (n = 6). It was observed that 87% (n = 59) of the patients with extensive metabolizer genotype (CC) exhibited high platelet inhibition as compared to 13% (n = 9) in the low response group (Table 2). With respect to the intermediate (CT) and poor metabolizer genotype (TT), the combined results showed that 77% or 40 of the 52 patients were in high response group while the rest demonstrated a low platelet inhibition response to clopidogrel (Table 2 and Fig. 1). These results suggest that there is no statistically significant difference between low and high responders to clopidogrel and studied genotypes.

**Figure 2:**
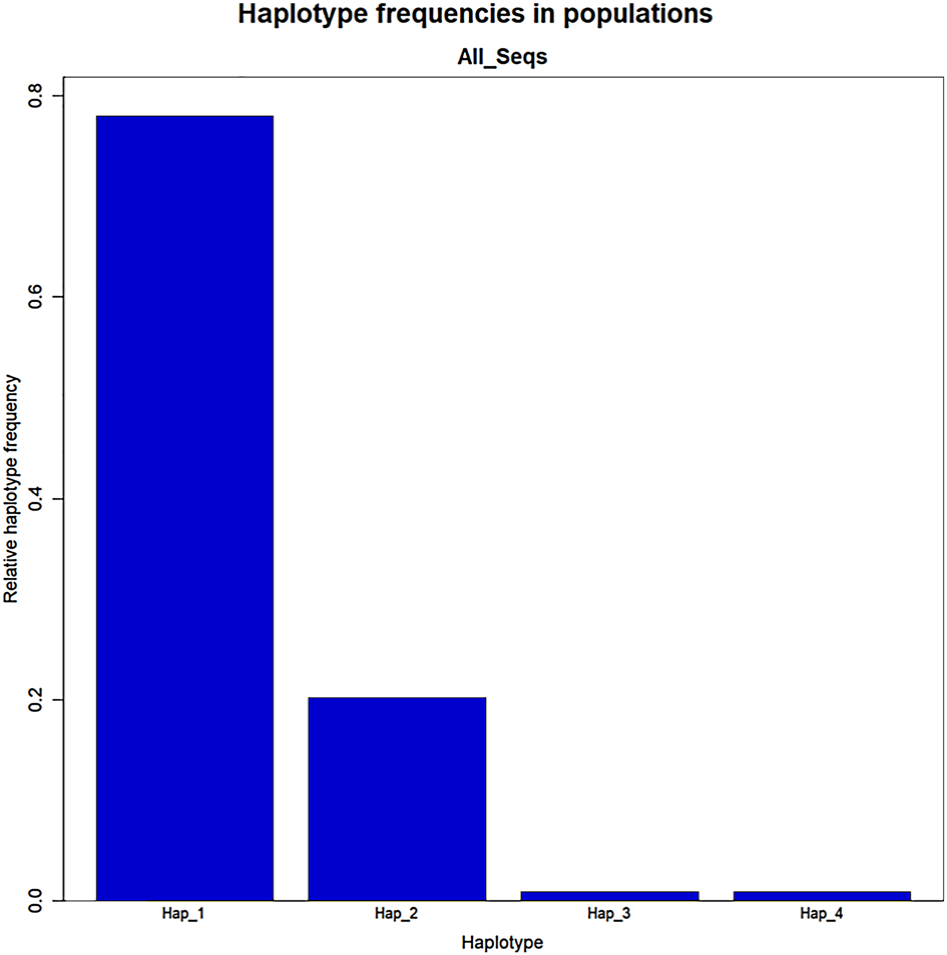
Haplotype frequency in *CYP2C19* of the Pakistani cohort.

CYP2C19*2 diplotype assignment was performed, and was categorized into 4 sub-haplotypes (denoted as H1-H4). Two haplotypes H1 and H2 were found with high frequency in this study. H1 was found with the highest frequency and was also associated with high antiplatelet response to clopidogrel (Table 2). It was observed that 48% (n = 10) of low responders had H1 haplotype while among the high responders, 77% (n = 76) had H1 haplotype. In patients exhibiting high response, 18% (n = 17) had H2 haplotype whereas among the low responders 24% patients (n = 5) possessed H2 haplotype. H3 and H4 were present with a minimum frequency of one each in our patient cohort (Fig. 2).

Correlations between antiplatelet response to clopidogrel and various laboratory and clinical laboratory parameters are presented in Table 3. A significant correlation (p = 0.025) was detected between the high clopidogrel responders and patient gender. No statistically significant correlation or significant difference was observed with regards to age, body mass index, concomitant diseases, and risk factors such as diabetes, hypertension, smoking, CAD familial history, and concurrent medications of the investigated patient cohort and the high responders to clopidogrel. A significant difference and a slight negative correlation were exhibited between leukocyte count and high responders to clopidogrel (R = − 0.206, p = 0.024). Furthermore, a significant correlation was established with regards to random glucose levels and high responders to clopidogrel (R = − 0.218, p = 0.017). No significant difference and no correlation could be established between high responders and the other remaining laboratory findings. The distribution of extensive, intermediate, and poor metabolizer genotypes was similar in high and low responder groups and no statistically significant correlation was found between the two groups in terms of these genotype. However, a negative correlation (R = − 0.233, p = 0.011) between the H1 haplotype and high responders to clopidogrel was observed. No such correlation was detected for the H2 haplotype.

**Table 3:** Correlation analysis between baseline variables and clopidogrel response.

Variable		Correlation co-efficient	p-value
Gender		0.204^a^	0.025*
Age		0.095^b^	0.302
BMI		0.040^b^	0.668
Concomitant diseases and risk factors	Diabetes	0.128^a^	0.160
	Hypertension	0.000^a^	1.000
	Smoking	0.072^a^	0.428
	Family history of CAD	− 0.047^a^	0.604
Indication	Acute coronary syndrome	0.187^a^	0.379
	Coronary artery disease		
	Congestive cardiac failure		
	Ischemic heart disease		
	Unstable angina		
Concurrent medication	Statin	0.113^a^	0.215
	ACE inhibitors	0.074^a^	0.419
	Low molecular weight heparins	− 0.001^a^	0.990
	Beta blocker	0.097^a^	0.290
Laboratory findings	PT (prothrombin time)	0.133^c^	0.148
	APTT (activated partial thromboplastin time)	0.077^c^	0.401
	Hemoglobin	− 0.107^c^	0.245
	White blood cells	− 0.206^c^	0.024*
	Platelets	0.134^c^	0.144
	Random blood sugar	− 0.218^c^	0.017*
	Urea	− 0.151^c^	0.100
	Creatinine	− 0.093^c^	0.310
	Fasting blood sugar	0.016^c^	0.862
CYP2C19*2 (3 groups)	CC	0.143^a^	0.291
	CT		
	TT		
CYP2C19*2 (2 groups)	CC	− 0.128^a^	0.160
	CT/TT		
Haplotype	H1	− 0.233^a^	0.011*
	H2	0.065^a^	0.475

*p-value ≤ 0.05.

^a^Phi correlation for categorical variables.

^b^Pearson correlation for normally distributed continuous variables.

^c^Spearman correlation for non-normally distributed continuous variables.

Several risk factors and variables were subjected to a bivariate regression analysis in order to elucidate the predictors of clopidogrel resistance (Table 4). Factors such as age, smoking status, and hypertension did not differentiate between low and high responders to clopidogrel while the presence of H1 haplotype, and diabetes mellitus were significant predictors of clopidogrel response (Table 4).

**Table 4:** Binary logistic regression analysis of risk factors for high platelet inhibition group.

Risk factor	Coefficient	Odds ratio	95% CI	p-value
Constant	− 0.661			0.516
Smoking	− 0.750	0.472	0.153–1.457	0.192
Hypertension	0.269	1.309	0.424–4.045	0.640
Diabetes	− 1.264	0.282	0.088–0.910	0.034*
Haplotype H1	1.427	4.164	1.453–11.936	0.008*
Age	0.034	1.035	0.985–1.087	0.175

*p-value ≤ 0.05.

**Figure 3:**
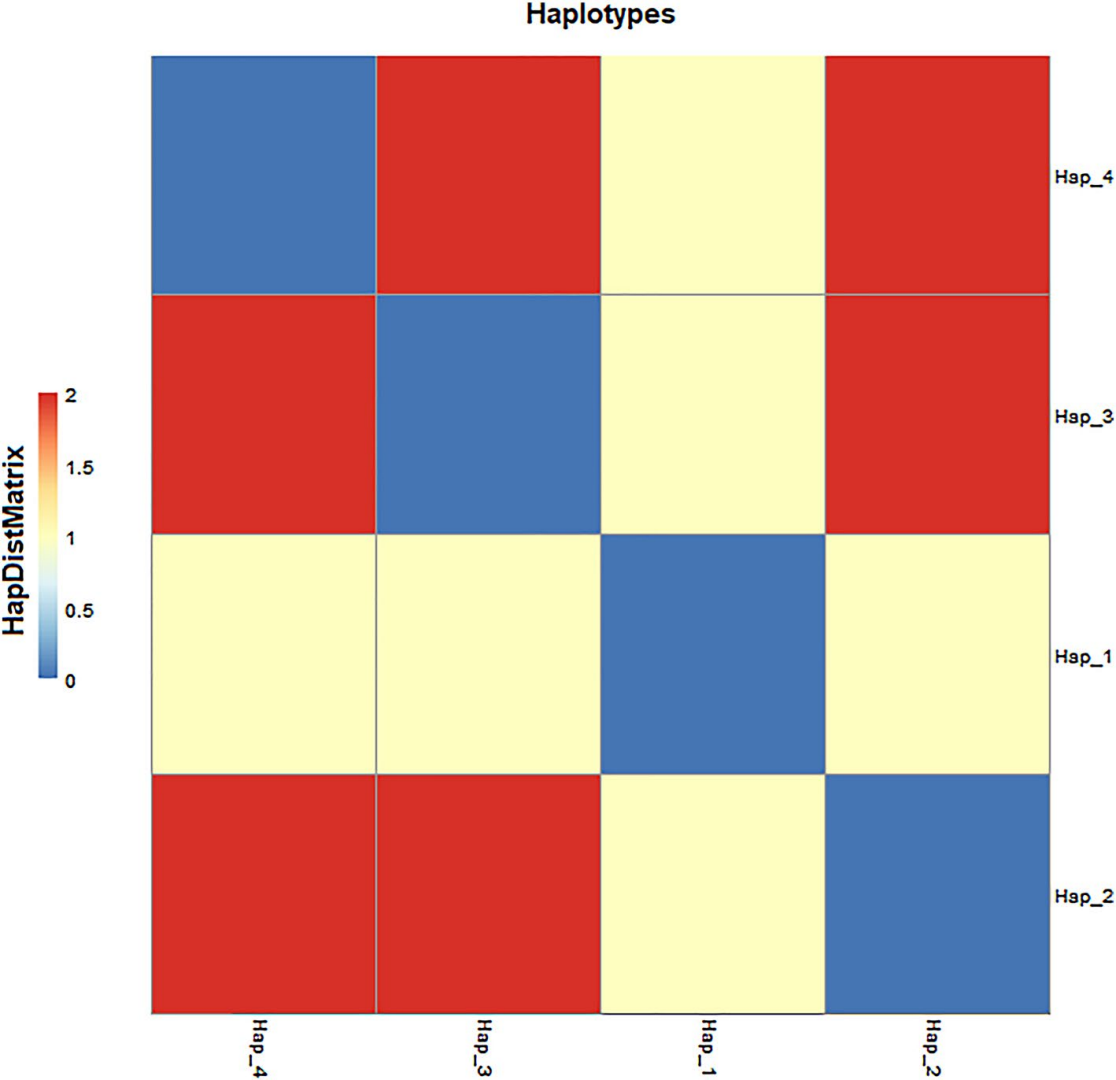
Pairwise difference of haplotypes among the Pakistani individuals of *CYP2C219* gene.

**Figure 4:**
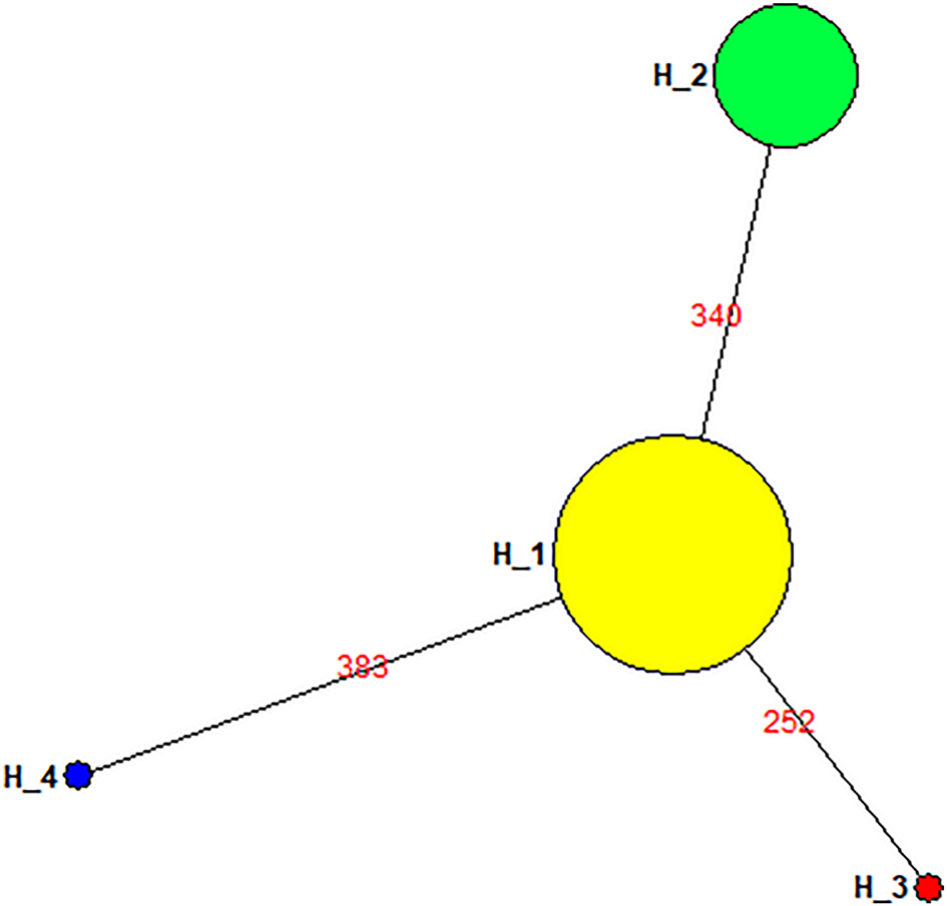
Haplotype-network of *CYP2C19* gene among Pakistani individuals: The area of the circle is proportional to the number of individuals holding that the specific haplotype. The line connecting to the node represent the genetic relatedness among Pakistani individuals.

As mentioned earlier, four haplotypes (H1-H4) with haplotype diversity (0.3542) were identified in the current study. The frequencies of these haplotypes are given in the Fig. 2. Due to the significant association observed between the high responders to clopidogrel and H1, haplotypes were investigated further. The pairwise genetic distances between haplotypes on the basis of AMOVA revealed that the H2 has substantial genetic differences against H3 and H4. On the other hand, H3 displayed high genetic distinction against H4, while H4 has significant distinction against H3 (Fig. 3). During the haplotype network analysis, the median-joining method revealed that H2 is a distinct individual group and highly distant form the H3 and H4 (Fig. 4).

## Discussion

The present study is the first of its kind that has investigated the relationship between clinical and demographical variables together with, more importantly, loss-of-function polymorphisms within the *CYP2C19* gene, and haplotypes and resistance to clopidogrel therapy in a Pakistani population. Previous studies throughout different regional and global populations exhibited a high variability when it comes to the prevalence of clopidogrel resistance, ranging from as low as approximately 5% to about 45% in German and Spanish populations respectively^25,26^. In the present investigation, 17.5% of patients in our cohort showed low response to clopidogrel. Wide interethnic variation in different populations such as from 20 to 65% in multiple populations in relation to the frequency of *CYP2C19*-reduced function genotypes has also been reported^27,28^. In our patient cohort, the frequency of the reduced function genotypes was 43%.

A total of 3 main *CYP2C19* genetic variants—*2, *3, and *17—with important influence on the bioavailability of clopidogrel, have been previously demonstrated^29^. The bioavailability of active clopidogrel in poor metabolizers is very low. Intermediate metabolizers, albeit having a reduced bioavailability, still exhibit inhibition of platelet activity, when administered with higher than normal doses of clopidogrel^30^. The majority of the population possess the (*1*1) alleles and are termed as extended metabolizers. Individuals possessing the (*1/*17) and (*17/*17) alleles referred to as rapid and ultra-rapid metabolizers respectively, have also been described, but we were unable to investigate *17 allele in the current study. Furthermore, the number of individuals possessing *17 allele in the general population is much smaller^30^.

In our investigation, there was no statistically significant correlation between the high and low responders to clopidogrel in terms of extensive, intermediate and poor metabolizer genotypes (Table 2). This might be due to the fact that we found no *3 allele in our genotyped samples, and a low prevalence of *2 allele. These findings are in line with previous reports^13^, where correlation between clopidogrel resistance and the status of intermediate metabolizers failed to reach statistical significance. Bhatt et al. proposed that the presence of loss-of-function allele could be linked to fewer bleeding complications but without a significant increase in the number of ischemic events^31^. However, our findings are slightly different from several previous studies^30,32^, some of which suggest that CYP2C19*2 allele is implicated in conferring resistance to clopidogrel therapy in multiple populations.

In the present study, we observed that low responder group had a significantly higher leukocyte count. This finding is in accordance with recent publications. Osmancik et al. reported that for individuals having a higher leukocytic count, there were increased incidences of high on treatment platelet reactivity^33^. It seems that the inflammatory mediators such as C-reactive protein, P-selectin, platelet soluble CD40 ligand, and interleukin 6, are able to enhance thrombocyte reactivity^33^. Interestingly, it has been postulated that high leukocyte counts could, in part, be responsible for conferring the controversial aspirin resistance as well^34^. Thrombocyte count and clopidogrel response were not significantly correlated in our cohort. These findings are in line with previous studies^35,36^.

Baseline characteristics indicated a slight difference in creatinine levels between the two groups; however, the difference was insignificant. Previous studies have also linked a wide variety of laboratory and clinical parameters to altered patient responses to clopidogrel therapy. Reduced thrombolytic activity has been observed in individuals diagnosed with and suffering from type 2 diabetes mellitus^37,38^. On the other hand, lower values of hematocrit have been related to high platelet reactivity^39^. Our data set shows that, apart from diabetes mellitus, none of these factors have significant difference in both low and high responders to clopidogrel. Similar results have been reported previously^40,41^.

Regarding concomitant administration of hydroxy methyl glutaryl CoA reductase inhibitors (statins), we found no difference, in terms of clopidogrel resistance, between those on or without such treatment. This is in line with most of the published data^42,43^. However, it is important to note that detrimental effects of statin treatment (with special consideration to those metabolized by CYP3A4) have been suggested^44,45^. A residual high on-treatment platelet reactivity was observed with the co-administration of rosuvastatin, which is chiefly metabolized by CYP2C9 with little involvement of CYP3A4^46^. It appears that the data obtained from ex vivo studies disagree with each other, suggesting different levels of interaction. Also, a clinically relevant finding is yet to be reported. Henceforth, it would be safe to suggest that a possible decreased clopidogrel response as a result of statin use, could have a significant impact particularly in high-risk patients.

It is well-known that cigarette smoking induces CYP1A2 enzyme which is responsible for the initial activation of clopidogrel^47^. Therefore, smoking status of a patient may affect antiplatelet response to clopidogrel. The explanation put forth for this smokers’ ‘paradox’ is that by enhancing the first step in the conversion of clopidogrel, the ‘induced’ CYP1A2 enhances the availability of active metabolites, thus decreasing the proportion of pro-drug excreted without activation^47^. However, in the present study, no association was found between the smoking status of the patients and the antiplatelet response to clopidogrel (Table 4).

Limitations of our study are that only ‘associations’ could be observed due to its cross-sectional design. Relevant survival profiles could not be created as the lack of clinical outcome data for most of the patients prevented us from doing so. The small sample size for the patients may have lowered the study’s statistical power. Concentrations of clopidogrel and its active metabolite within the plasma were not detected in the present study; thus, individual interviews were used to assume the correct administration of the drug.

The main findings of this study can be summarized as follows: low antiplatelet response to clopidogrel was found in 17.5% of our patients, and more than 43% of the patient population investigated had genotypes for the reduced function CYP2C19 enzyme. Carrying one or both variant alleles of CYP2C19*2 does not appear to significantly enhance the risk of having low response to clopidogrel. H1, the most frequent of the haplotypes, was associated with higher antiplatelet response with respect to clopidogrel. Low antiplatelet response to clopidogrel was associated with a higher leukocyte count. Higher random glucose levels were associated with lower antiplatelet response. Statins, beta-blockers or ACE inhibitor treatment, body mass index, or hematocrit levels didn’t significantly influence clopidogrel response in our data set but patients with diabetes mellitus did show differential response to clopidogrel treatment. Future studies, ideally with larger population samples, should investigate and assess additional clinical parameters and genomic mutations to evaluate and better foresee clopidogrel resistance.

## Material and method

### Data collection

The Institutional Review Board and Ethics Committee of Shifa Tameer-e-Millat University, Islamabad, Pakistan sanctioned the study. All the methods and procedures were performed in accordance with the relevant guidelines and regulations. All individuals were mandated to provide an informed consent to partake in the investigation. The study cohort comprised of 120 patients taking 75 mg clopidogrel at the time of inclusion in the study. Five milliliters venous blood was taken in the EDTA tube and stored at 4 °C. Gen Jet Genomics DNA extraction Kit (ThermoScientific) was used to isolate the genomic DNA. A 1% agarose gel electrophoresis was used to quantify the genomic DNA. Genomic DNA was stored at − 20 °C and was used later for further processing.

### Sequences and PCR information

From purified genomic DNA, the *CYP2C19* region containing significant and important variants was targeted for sequencing. The PCR products were purified and submitted to Tsingke Biotechnology, China, for commercial DNA sequence services. For sequence validation, each of the samples was sequenced into triplicates (3×). Data presented in the results contains variations on the reverse strand.

### Quality checking and filtration

The accuracy of the DNA sequences was assessed by the Staeden package and Finch. TV v1.4 (Geospiza, Inc; Seattle, WA, United States). High quality of the sequences has been assembled by the Lasergene package v1.7 (DNASTAR, Inc; USA).

### Genetic structure analysis

The good quality sequences of *CYP2C19* were aligned with the Reference Human Genome (GRch38)^48^. The SNP positions were identified against the Reference Genome. In Addition, the DnaSP v6 package^49^ was used to determine the haplotypes in the test sequences . The population statistical analyses like haplotypes distances and frequencies within the individuals, were performed by the Arlequin software v1.3^50^. The network of the haplotypes was drawn by the NETWORK v10 package^51^ using the median-joining method.

### Platelet aggregation

Whole blood aggregometry was used to investigate antiplatelet activity where ADP as platelet aggregation inducing agonist^52^. Within 30-min to 5-h of placing the blood samples in clean test tubes, comprising of 3.2% sodium citrate anticoagulant (9:1), patient blood samples were tested at a temperature of 37 °C with 1200 rpm stirring speed. Following manufacturer guideline, equivalent amount (volume) of normal saline was used to dilute 500 µL of citrated blood. The response (platelet aggregation) was recorded after placing the electrode in the cuvette where platelet aggregation was induced by ADP (10 µM). For up-to 6 min, recordings were obtained as a measure of electrical impedance in ohms. Percent mean platelet inhibition, after obtaining values from 3 to 4 individual experiments, was calculated.

### Statistical analysis

All data were recorded and analyzed using IBM Statistical Package for Social Sciences SPSS, version 23.0. Categorical variables were presented as frequency and percentage whereas for continuous variables, mean and standard deviation or median and interquartile range were calculated for normally distributed and non-normally distributed variables respectively. Normality was tested using Shapiro–Wilk test.

The study participants were categorized on the basis of their response to clopidogrel; maximal platelet aggregation of greater than 50% was characterized as low response and lower than 50% as high response. Comparison of the baseline variables (continuous) via independent samples T-test (or Mann–Whitney U test for non-normally distributed continuous variables) was performed for both response groups. For comparison of categorical variables, chi-square test (or Fisher exact test where expected count per cell is less than 5) was applied.

Correlation analysis was performed in order to study the association between independent variables and platelet inhibition. Pearson correlation (or Spearman’s rank correlation for non-normally distributed continuous variables) was applied to study correlation between continuous independent variables and percentage platelet inhibition. For categorical variables, phi correlation coefficient was calculated taking high response and low response groups as dependent variable. p ≤ 0.05 was regarded as statistically significant.
